# Controlling serum uric acid using febuxostat in cancer patients at risk of tumor lysis syndrome

**DOI:** 10.3892/ol.2014.2394

**Published:** 2014-07-30

**Authors:** MIHOKO TAKAI, TAKAHIRO YAMAUCHI, KEI FUJITA, SHIN LEE, MIYUKI OOKURA, SHINJI KISHI, YOSHIMASA URASAKI, AKIRA YOSHIDA, HIROMICHI IWASAKI, TAKANORI UEDA

**Affiliations:** 1 Department of Hematology and Oncology, University of Fukui, Matsuoka, Eiheiji, Fukui 910-1193, Japan; 2 Division of Infection Control, University of Fukui, Matsuoka, Eiheiji, Fukui 910-1193, Japan

**Keywords:** tumor lysis syndrome, febuxostat, hyperuricemia, hematological malignancy

## Abstract

Tumor lysis syndrome (TLS) is a life-threatening oncological emergency, in which control of serum uric acid (S-UA) levels is important. S-UA-lowering efficacy of a new xanthine oxidase inhibitor, febuxostat, was retrospectively evaluated in seven patients with hematological malignancies who were at an intermediate risk of developing TLS. A 10-mg dose of febuxostat was initiated and chemotherapy was started within 24 h of administering the first dose of febuxostat. Febuxostat was continued until at least day 7 of chemotherapy treatment. The UA-lowering treatment was considered effective if febuxostat reduced S-UA levels to ≤7.5 mg/dl by day 5. The mean S-UA level at base line was 6.4±2.6 mg/dl and, on day 5, the mean S-UA level was 4.7±1.8 mg/dl. All the patients achieved S-UA levels ≤7.5 mg/dl. Serum creatinine levels decreased from 0.93±0.25 to 0.85±0.25 mg/dl. The estimated glomerular filtration rate values increased from 69.7±24.5 to 76.9±26.2 ml/min. No adverse reactions were noted during the study period and no patients experienced progressive TLS. Successful control of S-UA and improved renal function were obtained in response to febuxostat treatment in cancer patients at a risk of TLS.

## Introduction

Tumor lysis syndrome (TLS) is a metabolic impairment that arises in cancer patients, and is caused by the release of cellular components into the bloodstream following the rapid lysis of cancer cells (1–3). TLS occurs most frequently in patients with hematological malignancies, such as acute lymphoblastic leukemia and Burkitt’s lymphoma, following the initiation of induction chemotherapy. TLS can also occur spontaneously in the context of any cancer associated with high proliferation and/or a large tumor burden (1–4). The release of intracellular contents, including nucleic acids, proteins, phosphorus and potassium, can lead to hyperuricemia, hyperkalemia, hyperphosphatemia, hypocalcemia and, subsequently, renal failure, arrhythmias, seizures and mortality (1–5).

Prevention and prompt treatment of TLS are crucial; therefore, early recognition of patient condition and evaluation of the risk of developing TLS is important. Guidelines and recommendations for the evaluation of risk of and prophylaxis against TLS have been published (2,3). TLS is divided into laboratory TLS and clinical TLS (2,3,6), for which Cairo and Bishop (6) developed a system based on modifications to the Hande-Garrow classification (7). Laboratory TLS is defined as two or more laboratory changes [such as serum uric acid (S-UA), potassium, phosphate and calcium levels] within 3 days before or 7 days after cytotoxic therapy (2,3). Clinical TLS requires the presence of laboratory TLS in addition to one or more of the following significant clinical complications: Renal insufficiency, cardiac arrhythmias, seizures or mortality. Risk of TLS varies with cancer type, tumor burden, renal function and laboratory findings, and can be categorized into low-, intermediate- and high-risk groups (2,3).

The primary aims of the prophylaxis against and direct management of TLS are to control serum concentrations of UA, potassium, phosphate and calcium, and to avoid the development of renal failure (8), of which lowering the S-UA is considered to be the most important. Hyperuricemia results from a rapid catabolism of purine-containing nucleic acids from tumor cells, since purine nucleic acids are converted to hypoxanthine, xanthine and, finally, to UA by xanthine oxidase. According to published guidelines (2,3), patients with an intermediate risk of developing TLS may be treated with allopurinol, while patients with a high risk of developing TLS may be given the recombinant uricase, rasburicase. Allopurinol is an hypoxanthine analog and an inhibitor of xanthine oxidase, which converts hypoxanthine to xanthine, and xanthine to UA (9). The action of allopurinol is relatively slow, taking several days to produce a reduction in S-UA levels. Furthermore, allopurinol is associated with various toxicities, including hypersensitivity reactions and hepatic damage (10). Notably, cancer patients receiving chemotherapy often exhibit renal dysfunction, which may increase the toxicity of allopurinol, because allopurinol and its metabolite, oxypurinol, are excreted by the kidney (11,12).

Febuxostat is a novel nonpurine-structured selective xanthine oxidase inhibitor, which may be a promising alternative to allopurinol in patients who are unable to tolerate allopurinol or in those with renal dysfunction (11,13–15). The present study retrospectively evaluated the UA-lowering efficacy of febuxostat in patients with hematological malignancies who were undergoing chemotherapy and at an intermediate risk of developing TLS. Evaluation measures focused on the reduction of S-UA, the management of TLS and the prevention of renal damage.

## Patients and methods

### Patients

Patients who were admitted to the University of Fukui Hospital (Fukui, Japan) between September 2011 and May 2012 were retrospectively evaluated in the present study. All the patients were newly diagnosed as having hematological malignancies and were at an intermediate risk of developing TLS. These patients received 10 mg febuxostat daily (the recommended starting dose for patients with gout and hyperuricemia) during the administration of induction chemotherapy. Patients did not receive any other medications that might otherwise affect S-UA levels, such as losartan, fenofibrate, atorvastatin, pyrazinamide or cyclosporine. This study was approved by the ethics committee of the University of Fukui Hospital.

### Risk classification

Risk classification for TLS was made based on the published guidelines (2,3). Diseases associated with an intermediate risk of TLS included acute myeloid leukemia with a peripheral white blood cell count between 10,000–50,000/μl; diffuse large B-cell non-Hodgkin’s lymphoma; and any other diseases associated with factors that could increase the risk of developing TLS, including, elevated serum lactate dehydrogenase, extensive bone marrow involvement, pre-existing renal disease or reduced urinary output (2,3).

### Categorization of hyperuricemia

Hyperuricemia is broadly classified into the following three types, UA-overproduction type, UA-underexcretion type and combined type, according to the guidelines for the Management of Hyperuricemia and Gout, published in Japan in 2010 (16). Urinary UA excretion, UA and creatinine clearance rate were determined to allow for categorization of the hyperuricemia type (urinary UA excretion >0.51 mg/kg/h and UA clearance rate >7.3 ml/min for overproduction type; urinary UA excretion <0.48 mg/kg/h or UA clearance rate <7.3 ml/min for underexcretion type; UA excretion >0.51 mg/kg/h and UA clearance rate <7.3 ml/min for combined type).

### Administration of febuxostat

All patients received 10 mg febuxostat orally after breakfast, once daily. Induction chemotherapy was initiated within 24 h of administering the first dose of febuxostat. Japanese insurance coverage approved the following administration schedule for febuxostat for patients with gout and hyperuricemia: 10 mg for the initiating dose, and 40–60 mg for maintenance doses. The administration of febuxostat was continued for ≥7 days.

### Assessments

The primary endpoint was the reduction of S-UA. Febuxostat treatment was considered to be successful, and the patient considered to be a treatment responder, if S-UA levels decreased to ≤7.5 mg/dl by day 5 of chemotherapy, according to previous studies (17,18). S-UA and serum creatinine (S-Cr) levels were determined in-house using a TBA-c16000 automatic analyzer (Toshiba Medical Systems, Tochigi, Japan) (19). Secondary endpoints included renal function and adverse events. Renal function was determined by S-Cr and estimated glomerular filtration rate (eGFR). Adverse events were evaluated for 10 days from the initiation of febuxostat according to the National Cancer Institute Common Terminology Criteria for Adverse Events 4.0 (May 28, 2009).

### Statistical analyses

All statistical analyses were performed using Microsoft Excel 2007 software (Microsoft Corporation, Redmond, WA, USA). All graphs were generated using GraphPad Prism software (Version 5.0; GraphPad Software, Inc., San Diego, CA, USA).

## Results

### Patient characteristics

Seven patients, who were admitted to the University of Fukui Hospital between September 2011 and May 2012, were evaluated retrospectively (Table I). The median patient age was 70 years (range, 36–79 years), and the study population consisted of six males and one female. The diagnoses of the patients included diffuse large B-cell lymphoma (n=2), acute myeloid leukemia (n=3), chronic myelomonocytic leukemia (n=1), and chronic myeloid leukemia (n=1). One patient (patient no. 6) already exhibited clinical TLS, and all other patients were at an intermediate risk of developing TLS (Table I).

### UA-associated parameters

The parameters associated with UA, which included the urinary UA excretion and UA clearance, were determined in the three patients with hyperuricemia (patient nos. 1, 6 and 7) (Table II). Based on the criteria for the Management of Hyperuricemia and Gout (16), four cases were classified as UA overproducers and three cases were classified as UA underexcretors (Table II). A previous study revealed that half of the patients with hyperuricemia with hematological malignancies were of the underexcretion type, although S-UA should still increase as a consequence of overproduction through tumor lysis (20).

### Therapeutic efficacy

**Figure 1:**
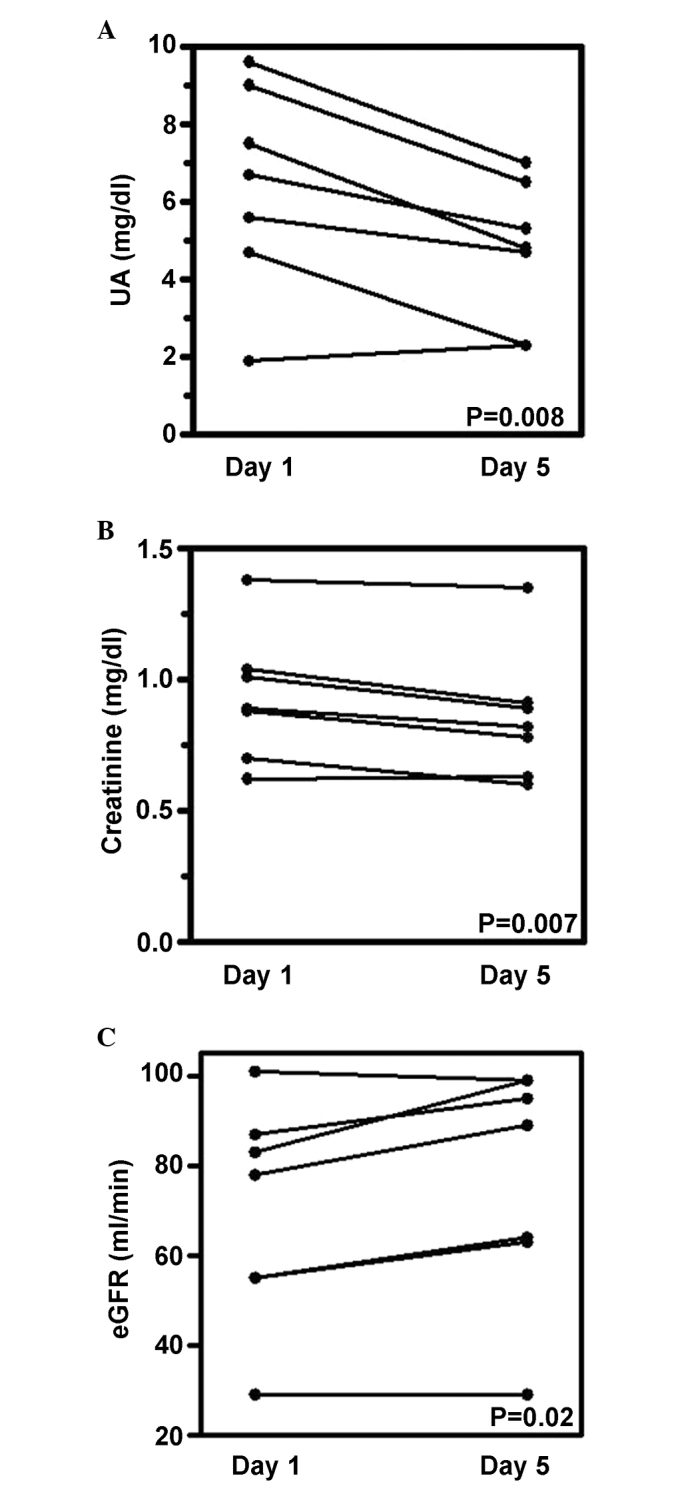
Serum (A) uric acid (UA) and (B) creatinine levels, and (C) estimated glomerular filtration rate (eGFR) at base line and on day 5 of chemotherapy.

The primary estimate of the present retrospective study was the S-UA reduction to ≤7.5 mg/dl by day 5 of chemotherapy treatment. While the S-UA level at base line was 6.4±2.6 mg/dl, S-UA on day 5 was 4.7±1.8 mg/dl (27% reduction; paired t-test, P=0.008) (Fig. 1A). All patients achieved S-UA levels ≤7.5 mg/dl by day 5. One patient (patient no. 2) had very low S-UA levels and the UA level was almost unchanged (from 1.9 to 2.3 mg/dl) (Fig. 1A). Assessment of the UA-associated parameters in this patient indicated the overproduction of UA, which was compensated for by simultaneous urinary UA overexcretion (Table II). This suggested that the effect of febuxostat may be masked by UA overexcretion. In terms of the secondary endpoints, S-Cr levels decreased from 0.93±0.25 to 0.85±0.25 mg/dl (paired t-test, P=0.007) (Fig. 1B). eGFR values increased from 69.7±24.5 to 76.9±26.2 ml/min (paired t-test, P=0.02) (Fig. 1C). No patient exhibited progression of TLS. These results suggested that febuxostat successfully controlled S-UA and improved renal function during chemotherapy.

### Adverse events

All adverse events that occurred during the 10 days of febuxostat administration are summarized in Table III. Grade 3/4 events, including cytopenia and sepsis, may be attributed to the concomitant administration of anticancer agents. Nausea and decreased appetite may also be as a result of the chemotherapy treatment. There were no indicators of grade 3/4 hepatic or renal dysfunction or attacks of gout; therefore, no severe adverse events specific to febuxostat appeared to occur.

## Discussion

Previously published guidelines (2,3) have stated that allopurinol and the recombinant uricase, rasburicase, are appropriate treatments for patients at intermediate and high risk of developing TLS. The hypoxanthine analog, allopurinol, is a traditional, competitive xanthine oxidase inhibitor that has been used for >40 years for the treatment of gout and hyperuricemia. The increase in S-UA observed in patients with TLS usually results from the catabolism of purine nucleic acid released from cancer cells upon cell lysis. It is considered to be effective in blocking xanthine oxidase, which would usually convert purine metabolites (hypoxanthine and xanthine) to UA, thereby reducing its production (2,3,8). Allopurinol, however, is not an ideal therapeutic agent (8), as it has slow onset of action (24–72 h) and is ineffective against already formed UA (21,22). Side effects occur in 3% of patients receiving allopurinol (23–25). Although allopurinol is generally safe and effective, it can induce life-threatening rashes and/or severe multisystem allopurinol hypersensitivity syndrome (12,26,27). Furthermore, the prolonged half-life (14–26 h) of the major allopurinol active oxidation product, oxypurinol, and its further prolongation in patients with decreased creatinine clearance necessitates dose reduction to avoid the side effects and severe toxicities. This dose reduction can attenuate the UA-lowering efficacy (12).

Febuxostat is a UA-lowering agent used for the treatment of patients with gout and hyperuricemia. Febuxostat is a non-purine-structured xanthine oxidase inhibitor that is thought to be independent of other enzymes in the purine and pyrimidine metabolic pathways (13–15). Febuxostat is primarily metabolized in the liver, and is eliminated by both the hepatic and renal pathways, equally (13–15,28). Febuxostat has been extensively studied in clinical trials involving patients with gout (13–15,29). In the CONFIRMS trial, 2,269 patients were randomized to 40 or 80 mg febuxostat or 300 mg allopurinol. The endpoints included the proportion of all subjects with S-UA levels <6.0 mg/dl, and the proportion of subjects with mild/moderate renal impairment and S-UA levels <6.0 mg/dl. The study revealed that the UA-lowering efficacy of febuxostat at an 80 mg dose exceeded the efficacy produced by treatment with allopurinol. Furthermore, in subjects with mild/moderate renal impairment, both febuxostat doses were more effective than and were equally safe as allopurinol (29). As a result of the demonstrated efficacy and safety, febuxostat was used in the present study as an alternative to allopurinol in cancer patients at risk of TLS.

The present retrospective study revealed that febuxostat at 10 mg sustained or reduced S-UA to levels ≤7.5 mg/dl during chemotherapy. In Japan, the maintenance dose of febuxostat is 40–60 mg/day, and the starting dose is 10 mg. Therefore, patients in the present study were given a 10-mg dose, which despite at this low dose level, achieved a 27% reduction in S-UA within one week. The use of febuxostat at maintenance doses (40–60 mg) has a greater potential for a higher efficacy in the control of hyperuricemia associated with TLS.

A low starting dose of febuxostat is often prescribed to reduce the risk of acute flares of gout associated with a steep decrease in S-UA. Attacks are triggered by urate crystal mobilization with a lowering of S-UA by febuxostat. A more abrupt lowering with febuxostat would induce gout more frequently as compared with allopurinol (12). Hyperuricemia in cancer patients at a risk for TLS have not been reported to commonly experience flares of gout (20,30). This may be attributed to the short duration of a hyperuricemic state in these patients, which is unlikely to generate urate crystal deposition.

In conclusion, the present retrospective study has demonstrated that febuxostat was safe and effective in preventing or reversing hyperuricemia in patients with hematological malignancies, who were undergoing chemotherapy and at an intermediate risk of developing TLS. A prospective study is undergoing to confirm the efficacy of febuxostat for the prevention and treatment of hyperuricemia in cancer patients at risk of TLS.

## Figures and Tables

**Table I:** Patient’s characteristics.

Patient no.	Age (years)	Gender	Diagnosis	WBC (/μl)	LDH (U/l)	S-UA (mg/dl)	S-Cr (mg/dl)	S-Ca (mg/dl)	S-K (mg/dl)	S-P (mg/dl)	eGFR (ml/min/1.73 m^2^)	LTLS/CTLS
1	53	M	DLBCL	11,500	159	7.6	0.89	8.8	4.1	2.8	87	(−)/(−)
2	59	M	AML	25,400	356	1.9	0.62	8.4	3.6	3.2	101	(−)/(−)
3	74	M	AML	42,100	745	5.6	0.70	8.8	3.5	3.9	83	(−)/(−)
4	36	M	AML	3,200	195	4.2	0.88	8.3	3.5	2.9	78	(−)/(−)
5	76	M	DLBCL	5,800	237	6.2	1.01	9.2	4.7	3.7	55	(−)/(−)
6	79	F	CMML	60,700	302	9.6	1.38	9.5	3.8	4.6	29	(+)/(+)
7	70	M	CML	347,000	829	9.0	1.04	9.3	4.6	3.7	55	(−)/(−)

M, male; F, female; DLBCL, diffuse large B-cell lymphoma; AML, acute myeloid leukemia; CMML, chronic myelomonocytic leukemia; CML, chronic myeloid leukemia; WBC, white blood cell (normal range, 3400–9600/μl); LDH, lactate dehydrogenase (normal range, 119–214 U/l); S-UA, serum uric acid (normal range, 2.6–7.0 mg/dl); S-Cr, serum creatinine (normal range, 0.46–0.78 mg/dl); eGFR, estimated glomerular filtration rate; LTLS, laboratory tumor lysis syndrome; CTLS, clinical tumor lysis syndrome.

**Table II:** Parameters associated with uric acid.

Patient	U-UA (mg/kg/h)	CUA (ml/min)	Type of hyperuricemia
1	0.36	5.1	Under-excretion
2	0.52	35.2	Overproduction
3	0.86	15.1	Overproduction
4	0.25	11.0	Under-excretion
5	0.24	3.8	Under-excretion
6	0.80	8.3	Overproduction
7	2.40	34.0	Overproduction

U-UA, urinary uric acid excretion (normal range, 0.483–0.509 mg/kg/h); CUA, uric acid clearance (normal range, 7.3–14.7 ml/min).

**Table III:** Adverse events following the administration of febuxostat.

Adverse event	All grades, n	Grade 3/4, n
Constipation	5	0
Hyperglycemia	4	1
White blood cells decreased	3	3
Neutrophil count decreased	3	3
Thrombocytopenia	3	3
Diarrhea	3	0
Decreased appetite	2	0
Nausea	2	0
Bilirubin increased	2	0
Hemoglobin decreased	1	1
Sepsis	1	1
Increased aspartate aminotransferase	1	0
Increased alanine aminotransferase	1	0
Fatigue	1	0
Peripheral edema	1	0
Hyponatremia	1	0
Vomiting	1	0
Stroke	1	0

Adverse events were evaluated for 10 days from the initiation of febuxostat according to the National Cancer Institute Common Terminology Criteria for Adverse Events 4.0 (May 28, 2009).

## Abbreviations

TLS: tumor lysis syndrome
UA: uric acid
S-UA: serum uric acid
S-Cr: serum creatinine
eGFR: estimated glomerular filtration rate
